# Point-of-Care Diabetes Diagnostics: Towards a Self-Powered Sensor

**DOI:** 10.3390/mi16020134

**Published:** 2025-01-24

**Authors:** Inês Vinagre, Gabriela V. Martins, Joaquim A. Alves, Felismina T.C. Moreira

**Affiliations:** CIETI-LabRISE, School of Engineering, Polytechnic Institute of Porto, 4200-072 Porto, Portugal; imdsv@isep.ipp.pt (I.V.); gfdvm@isep.ipp.pt (G.V.M.); jaa@isep.ipp.pt (J.A.A.)

**Keywords:** enzymatic fuel cell, glucose oxidase, glucose, biographene, Prussian blue nanocubes, biosensor, electrochemical detection

## Abstract

A cutting-edge biosensor has been developed to monitor blood glucose levels, which is particularly vital for people with diabetes. This advanced technology uses a miniaturized and membraneless enzymatic fuel cell (EFC) as a compact electrical reader for rapid on-site diabetes diagnosis. Using disposable screen-printed gold electrodes (Au-SPE) modified with the enzyme glucose oxidase (GOx), the biosensor enables the oxidation of glucose at both the anode (counter electrode) and cathode (working electrode) of the EFC. The cathode contains graphene oxide/Prussian blue nanocubes (GO/PBNCs), while the anode uses a biographene layer. Both electrodes were modified with GOx by electrostatic/hydrogen bonding the enzyme to the modified electrodes surface. Individual evaluations of each electrode system emphasized their effectiveness. The integration of both electrodes resulted in an EFC that can generate an output power of approximately 1.8 μW/cm^2^ at a glucose concentration of 5 mmol/L, which is very close to physiological conditions (3.8 to 6.9 mmol/L). This technology represents a significant advance and promises fully autonomous diagnostic devices suitable for a wide range of analytes. It paves the way for diagnostics everywhere and marks a fundamental shift in point-of-care (PoC) diagnostics.

## 1. Introduction

Enzymatic fuel cells (EFCs) are self-powered (bio)generators that harvest bioenergy from biochemical reactions in order to produce electricity using enzymes as the catalysts. This technology shows advantages in terms of mild operating conditions (temperature and pH) and potential applications as in vivo power sources [1,2,3,4,5]. The first commercial EFC based on glucose biosensor was manufactured by Kyoto Daiichi Kagagu in Japan in 1996 and marketed as the Glucocard [6,7]. Several works have been reported in the literature based on EFC sensors for glucose detection in biological samples [7,8,9,10,11,12]. These EFCs show several advantages. However, they still have some limitations, such as the requirement for two or more different enzymes, membranes, or mediators, which increases costs and decreases simplicity. Thanks to the above-mentioned advantages and their good biocompatibility, as well as their application in fields like implantable energy delivery devices and EFC-based biosensors, EFCs are attracting more and more attention from scientists all over the world [13,14]. Compared with conventional enzymatic electrochemical biosensors, these devices based on EFCs have two major advantages: they have a simple design with only two electrodes and do not require a battery or external energy source. Because they utilize the analyte as a fuel, they can power themselves and determine the concentration of the analyte at the same time. The two-electrode system and the lack of an external energy source greatly simplify the required electronics and enable the development of miniature EFC-based biosensors [15]. The sensor data of these biosensors can be transmitted wirelessly with a radio transmitter, enabling their use as implanted, remote, self-powered EFC-based biosensors for continuous monitoring of changes in the concentration of a particular analyte in the human body. EFCs have already been implanted in living organisms, such as cockroaches [16], snails [17], rats [18], and a vein replica [19]. It is likely that implanted EFCs will be used in the future to monitor blood glucose levels in diabetics and to detect various substances that cause cancer or heart disease [20]. The number of publications on EFCs is increasing year by year and includes analytes such as ascorbic acid [21], lactate [22], glutathione [23], acetylcholine [24], cholesterol [7], and carcinoembryonic antigen [25]. However, the greatest interest is in EFC-based glucose biosensors. This is not surprising, as more than 400 million people worldwide suffer from diabetes, a metabolic disease characterized by the body’s inability to control blood glucose levels. Monitoring and controlling blood glucose levels is essential to prevent serious complications of diabetes, such as heart attack, stroke, kidney failure, blindness, and lower limb amputation. According to the International Diabetes Federation [26], 285 million people worldwide have diabetes, and this number is expected to rise to 438 million by 2030, with 66.7% of diabetes cases occurring in developing countries [27,28,29,30]. Given these numbers, it is imperative to develop new tools for accurate, simple, cost-effective, and autonomous biosensing for the diagnosis of diabetes. Several point-of-care (PoC) devices have been developed for diabetes diagnosis [31,32,33,34], including colorimetric test strips based on lateral flow assays [35] and electrochemical biosensors [36,37,38]. These devices offer interesting advantages in terms of sensitivity, fast response time, portability, and power consumption.

Some research has described a battery-powered biosensor for monitoring cholesterol that uses cholesterol oxidase as a simple enzyme to oxidize the substrate. In the cathode, hydrogen peroxide is reduced by the action of Prussian blue, and in the anode, the product of the reaction is oxidized by a mediated reaction with phenotiazine for the same enzyme [7]. Another interesting work with glucose oxidase (GOx) in the anode or cathode was described in 2014 by [39]. Mediated reduction occurred at the anode by 5-amino-phenanthroline and at the cathode by horseradish peroxidase. This research is very noteworthy; however, the electrocatalysis at the cathode is based on a bi-enzymatic assay and uses mediators at both electrodes, making the assay more expensive and complex. There is also the possibility that the mediators are washed out into the solution after incubation with the substrate.

Here we report an innovative biosensor approach. The advance in this work lies in the development of a novel biosensor for monitoring blood glucose levels that uses a miniaturized, membraneless EFC for rapid on-site diabetes diagnosis. The device consists of a gold screen-printed electrode (Au-SPE) modified with glucose oxidase to facilitate glucose oxidation. The cathode incorporates graphene oxide/Prussian blue nanocubes (GO/PBNCs), while the anode employs a biographene layer. A key advance is the integration of sustainably produced nanomaterials, with the biographene serving as a raw material for the synthesis of graphene oxide, which is subsequently used to produce the GO/PBNC composite. This sustainable approach improves the environmental impact of the device while maintaining its diagnostic efficiency.

## 2. Materials and Methods

### 2.1. Reagents

All chemicals were of analytical grade and water was deionized or ultrapure Milli-Q laboratory grade. The following chemicals were used: phosphate buffered saline (PBS) tablets (Amresco, Solon, OH, USA); potassium chloride (Merck, Rahway, NJ, USA); Bovine Serum Albumin (BSA), potassium hexacyanoferrate III (K_3_[Fe(CN)_6_]), and Iron (III) chloride (FeCl_3_) from Fluka. Graphite power (99%) was purchased from Sigma (St. Louis, MO, USA) and glucose oxidase (GOx) from Sekisui Diagnostics (Burlington, MA, USA). Polyethyleneimine (PEI) was obtained from Aldrich (St. Louis, MO, USA). Glucose (Alfa Aesar), human serum (Cormay), and creatine were obtained from Sigma Aldrich (St. Louis, MO, USA).

### 2.2. Apparatus

The electrochemical measurements were conducted with a potentiostat/galvanostat from Metrohm Autolab (PGSTAT302N, Utrecht, The Netherlands). Au-SPEs (Oviedo, Spain) were purchased from DROPSENS (DRP-C250AT), with a working electrode made of gold, a counter electrode made of platinum, and a reference electrode and electrical contacts made of silver. The diameter of the working electrode was 4 mm, and the Au-SPEs were placed in a switch box (DROPSENS, Oviedo, Spain), interfacing the electrical contacts of the Au-SPE with the electrical connections of the potentiostat equipment. Transmission Electron Microscopy (TEM), (JEOL Ltd.) images were acquired on a JEOL JEM 1400 equipment at 120 kV (Tokyo, Japan). Scanning Electron Microscopy (SEM) was performed using the high-resolution (Schottky) microscope Quanta 400 FEG ESEM/EDAX Genesis X4M (FEI Europe B.V., Eindhoven, The Netherlands).

### 2.3. Electrochemical Procedures

The electrodes were characterized by cyclic voltammetry (CV) in phosphate buffered saline (PBS) buffer with 0.1 mol/L KCl. For the cathode tests, the potential was scanned from −0.1 to +0.5 V at 10 mV/s in buffer and in a 5 mmol/L solution of glucose. The electrochemical characterization of the anode was performed by CV in PBS buffer in a potential swept from 0.0 to +0.8 V at a rate of 5 mV/s in buffer and in a 5 mmol/L solution of glucose.

The calibration curve was performed by chronoamperometry (CA) with a constant applied potential of 0 and +0.4 V for the cathode and anode, in the concentration range of 0.5 to 50 mmol/L for the anode and 0.5 to 25 mmol/L for the cathode and anode. Approximately 80 µL of each standard was added to the electrode surface, and the electrochemical signal was monitored with the CA technique. All tests were performed in triplicate.

The accuracy tests were conducted by performing recovery assays on commercial serum samples spiked with glucose concentrations of 0.5 and 5 mmol/L, using an anode-based electrode. These assays were evaluated using the CA technique, applying a potential of +0.4 V for 120 s. The performance of the EFC was determined by linear sweep voltammetry (LSV) at a scan rate of 5.0 mV/s. The EFC parameters were determined using the anode as the working electrode and the cathode as the combined counter/reference electrode without a separating membrane. Power was evaluated using LSV, varying the voltage from the open circuit potential (OCP) to lower voltage ranges at a scan rate of 5.0 mV/s. All tests were performed in triplicate.

### 2.4. Electrodes Preparation

#### 2.4.1. Anode

Biographene (Bgr) was produced following reference [40]. A suspension of graphite crystals (100 mg/mL) in 200 mL of deionized (DI) water with a pH of 7.0 containing the BSA (3.0 mg/mL) was ground in a kitchen blender for 30 min. Samples were taken every 5 min to study the rate of exfoliation, and the blender was stopped to avoid overheating (<30 °C). The absorbance at 660 nm of the suspension was used to quantify the graphene concentrations. Afterwards, the unexfoliated graphite was separated by centrifugation at 1500 rpm for 45 min.

To build up the anode, the counter electrode of the Au-SPEs was modified with 3 layers of Bgr. About 5 μL of the suspension was dropped onto the Au-SPEs three times and dried at 37 °C. Subsequently, the enzyme (Sekisui Diagnostics, Kent, UK) GOx (20 mg/mL in PBS buffer pH 7.2) was incubated on the Bgr layer and dried overnight in a refrigerator Beko(Arçelik A.Ş., Istanbul, Turkey ) (4 °C). The characterization of the biosensor was performed in PBS (VWR International from Radnor, PA, USA) pH 7.2 solutions doped with 0.1 mol/L KCl.

#### 2.4.2. Cathode

The synthesis of the composite followed the procedure described previously [41]. A total of 10 mL of 5 mmol/L FeCl_3_.6H_2_O (pH 1.1), 1 mL of 3% PEI, and 10 mL of 5 mmol/L K_3_Fe(CN)_6_ (pH 1.1) were added to 10 mL biographene dispersion (6.7 mg/mL, pH 1.1) with stirring, then heated and refluxed for 3 h. During the reaction, the color of the mixture gradually changed from yellow to dark blue, indicating the formation of a composite of Prussian blue nanocubes and graphene oxide (PBNCs/GO). The final mixture was centrifuged and washed 3 times with ultrapure water and redispersed in 10 mL of water. Next, the Au-SPE was modified with a layer of PBNCs/GO composite casted onto the surface of the working electrode. The composite layer was cured overnight at 60 °C. The PBNCs/GO modified electrodes were pretreated by CV in 0.1 mol/L KCl with 0.1 mol/L HCl. CV was performed in the range of −0.2 to +0.5 V with a sampling rate of 50 mV/s for 50 cycles after stabilizing in 0.1 M KCl mixed with PBS buffer pH 7.2. The electrodes modified with GOx/PBNCs/GO were then obtained by incubating the enzyme (GOx, 20 mg/mL) in the working electrode of the Au-SPE and dried overnight in a refrigerator (4 °C).

### 2.5. Biofuel Cell SETUP

**Figure 1:**
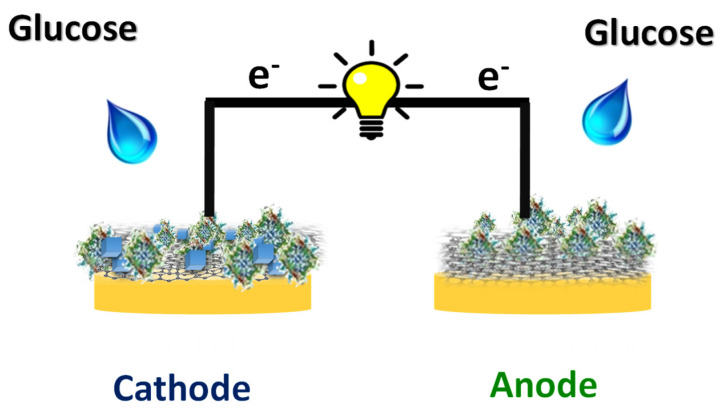
Representation of the EFC assembly. The enzyme GOx was immobilized in the working electrode (cathode) and in the counter electrode (anode).

A self-powered glucose biosensor was developed by combining GOx/PBNCs/GO as the cathode and GOx/Bgr modified Au-SPE as the anode. Polarization tests were performed using LSV with a sampling rate of 5.0 mV/s, from OCP to the lower voltage. The solution for the evaluation of the EFC consisted of 100 μL PBS buffer pH 7.4 containing different concentrations of glucose as fuel for the anode and cathode. The amperometric glucose biosensor based on an EFC, investigated and described in this work, was constructed by coupling an anode and a cathode, both modified with the same enzyme—GOx (Figure 1) in an Au-SPE.

Overall, the working surface of the Au-SPE acted as a (bio)cathode and was modified with the GO/PBNCs composite. Subsequently, the enzyme was adsorbed on its surface. The counter electrode of the Au-SPE, modified with Bgr and further Gox adsorbed on its surface, served as a (bio)anode. In the presence of glucose, the Bgr layer was able to transfer the electrons generated during GOx-catalyzed glucose oxidation from the redox-active prosthetic group of the enzyme to the surface of the (bio)anode through oxygen. At the same time, the PBNCs on the surface of the (bio)cathode showed electrocatalytic activity in the reduction of hydrogen peroxide generated on both bioelectrodes during the enzymatic reaction [41]. The combination of these two bioelectrodes made it possible to develop the glucose biosensor based on the single-enzyme biofuel cell of which the (bio)anode and (bio)cathode were powered by the same substrate—glucose.

## 3. Results

### 3.1. Qualitative Analyses of the Nanomaterial’s

**Figure 2:**
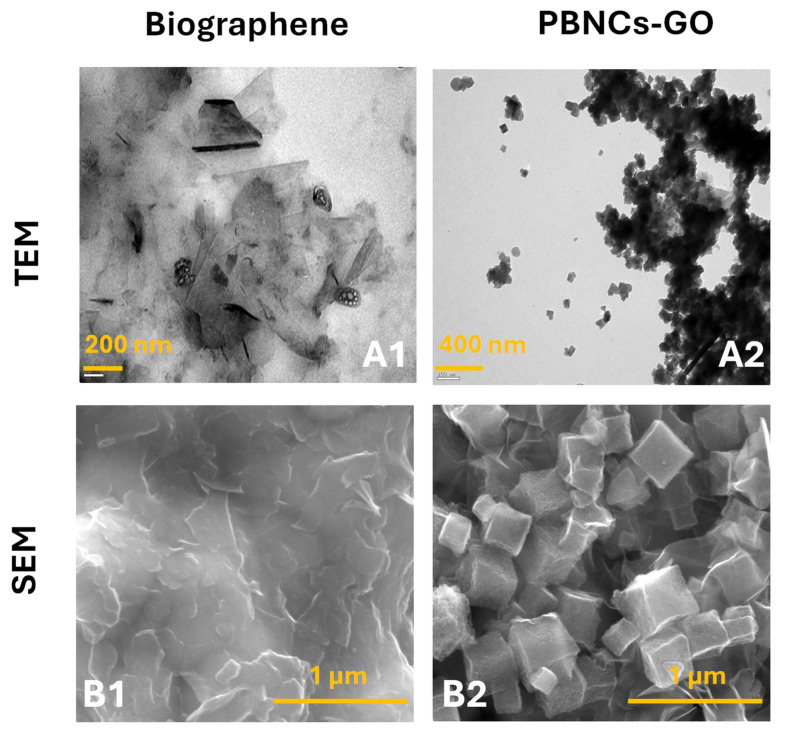
Results regarding TEM analysis of (**A1**) Bgr and (**A2**) PBNCs-GO nanomaterials; results regarding SEM analysis of (**B1**) Bgr and (**B2**) PBNCs-GO nanomaterials.

The morphological characterization of Bgr and PBNCs-GO was carried out using TEM and SEM analyses (Figure 2). The TEM images (Figure 2A1) clearly show the graphite sheets that make up Bgr with clearly visible dark spots under the microscope. These spots are caused by the collective stacking of graphene layers, which is characteristic of graphite-based materials. In Figure 2A2, the TEM image shows the modified biographene layers with incorporated PBNCs, confirming the successful modification of the Bgr with PBNCs.

The SEM image of the biographene (Figure 2B1) shows a morphology consistent with the TEM findings, confirming the presence of graphene layers. The SEM image shows the topography and surface structure of the material at higher magnification, with individual graphene layers clearly visible. For the PBNCs-GO nanocomposite, the SEM image shows a remarkable contrast revealing the presence of numerous cubic PB nanoparticles uniformly distributed over the reduced graphene oxide (rGO) nanosheets (Figure 2B2). These PB nanoparticles are very uniform in size, with an average size in the nanometer range. They are randomly distributed over the silk-like surface of the rGO nanosheets, demonstrating the successful integration of the PBNCs on the graphene oxide substrate. This arrangement indicates an efficient and controlled growth of the PBNCs on the surface of the reduced graphene oxide, leading to the formation of a high-quality PBNCs-GO nanocomposite.

### 3.2. Electrochemical Characterization of the Bgr- and PBNCs-GO-Based Biosensors

**Figure 3:**
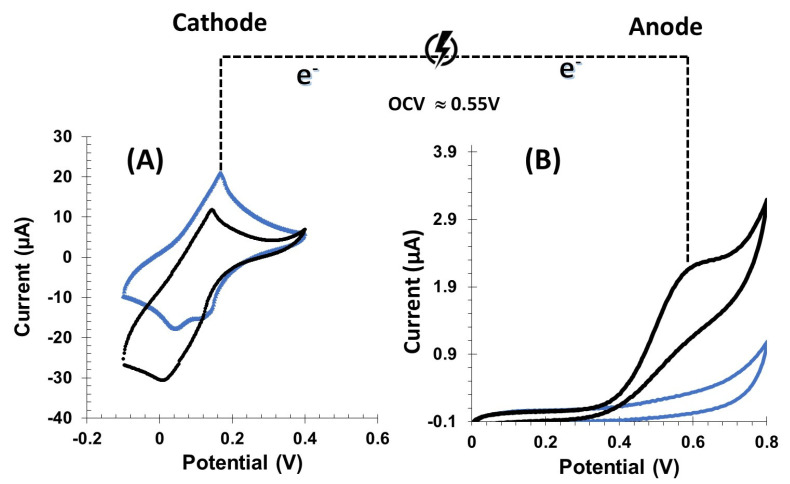
CV of differently modified electrodes in the potential range of the biocathode (**A**) and the bioanode (**B**). Cyclic voltammograms were performed in PBS buffer pH 7.2 containing 0.1 mol/L KCl (blue dots) and in glucose 5 mmol/L prepared in PBS buffer pH 7.2 containing 0.1 mol/L KCl at a scan rate of 10 mV/s (black dots).

Figure 3 represents the electrochemical cell (EFC) based on a GOx/Bgr anode and a GOx/PBNCs-GO cathode. The catalytic processes occurring at the cathode are similar to those described in previous studies [40]. At the GOx/PBNCs-GO-based cathode, glucose is oxidized by GOx, generating hydrogen peroxide (H_2_O_2_) as a product. The H_2_O_2_ produced is then reduced to water at the cathode, with the PBNCs acting as the electrocatalyst. This reduction process is facilitated by the PBNCs, which promote the electrochemical reduction of H_2_O_2_ to H_2_O, as previously reported in the literature [13,40,41,42,43]. At the anode, the oxidation of glucose occurs through the action of GOx, which generates gluconolactone and electrons

The (bio)electrochemical reactions are shown in Equations (1)–(4). The catalytic processes occurring on the cathode were similar to that stated by [41]. Equations (1) and (2) represent an oxygen-mediated GOx-catalyzed glucose oxidation based on a GOx/BGr anode. At the GOx/PBNCs-GO-based cathode, glucose is oxidized by GOx (Equations (1) and (2)), and then H_2_O_2_ is produced and reduced to water by the electrocatalyst PBNCs (Equations (3) and (4)). The amount of H_2_O_2_ produced is proportional to the amount of glucose consumed [13,40,41].

(1) $$GOx\left(ox\right)+Glucose\to GOx\left(red\right)+gluconolactone$$

(2) $$GOx\left(red\right)+O_{2}\to GOx\left(ox\right)+H_{2}O_{2}$$

(3) $${{Fe}_{4}}^{III}\;[{Fe}^{II}{\left({CN)}_{6}\right]}_{3}+4K^{+}+4e^{-}↔K_{4}\{{{Fe}_{4}}^{II}\left[{Fe}^{II}{\left({CN)}_{6}\right]}_{3}\right\}$$

(4) $$K_{4}\{{{Fe}_{4}}^{II}\left[{Fe}^{II}{\left({CN)}_{6}\right]}_{3}\right\}+2H_{2}O_{2}+{4H}^{+}\to {{Fe}_{4}}^{III}\;[{Fe}^{II}{\left({CN)}_{6}\right]}_{3}+4K^{+}+4H_{2}O$$

The functionality of the (bio)cathode (Figure 3A) and the (bio)anode (Figure 3B) were evaluated individually using CV in PBS buffer at pH 7.2 spiked with 0.1 mol/L KCl with or without the addition of 5 mmol/L glucose. After the addition of glucose, well-defined (bio)electrocatalytic currents were observed at both electrodes, confirming the enzymatic activity of GOx in the presence of glucose.

The electrochemical activity of bioanode was evaluated using CV both in the presence and absence of glucose. In the presence of glucose (Figure 3B, black curve), a small oxidation current was observed, starting at about +0.4 V and reaching a peak around +0.55 V. This current is associated with the oxidation of H_2_O_2_, a by-product of glucose oxidation by GOx. The occurrence of this peak indicates that H_2_O_2_, which is formed during glucose oxidation, is electrochemically oxidized at the anode. The oxidation of H_2_O_2_ is an important step for the performance of the bioanode, as it helps to generate the electron flow required for the overall electrochemical reaction. In the case of the biocathode, the addition of 5 mmol/L glucose to the PBS-KCl solution led to a rapid increase in the cathodic current. This increase is attributed to the reduction of H_2_O_2_ produced by the enzymatic reaction at the anode. The (bio)cathode showed a more negative net current when the CV was performed in the presence of glucose compared to the buffer alone, indicating the reduction of H_2_O_2_ at the cathode. This behavior confirms the electrocatalytic activity of the cathode, where PBNCs or other catalysts likely promote the electroreduction of H_2_O_2_ to water, a typical reaction at the cathode in biofuel cells.

The CV results thus show effective bioelectrocatalysis at both the anode and the cathode, with glucose oxidation and H_2_O_2_ reduction occurring efficiently at the respective electrodes. The biocathode reacts to the reduction of H_2_O_2_, while the bioanode is involved in glucose oxidation and the subsequent production of H_2_O_2_.

### 3.3. Analytical Performance of the Electrodes

#### 3.3.1. Bgr-Based Sensor

The calibration curve for glucose detection was generated using CA with a potential of +0.6 V applied for 120 s. Successively, 80 µL of each glucose solution, starting with the lowest concentration (between 0.05 and 50 mmol/L), was applied to the electrode surface. After collecting the CA data, the current values were recorded 2 min after the addition of each glucose concentration. These data were used to generate the respective calibration curves.

**Figure 4:**
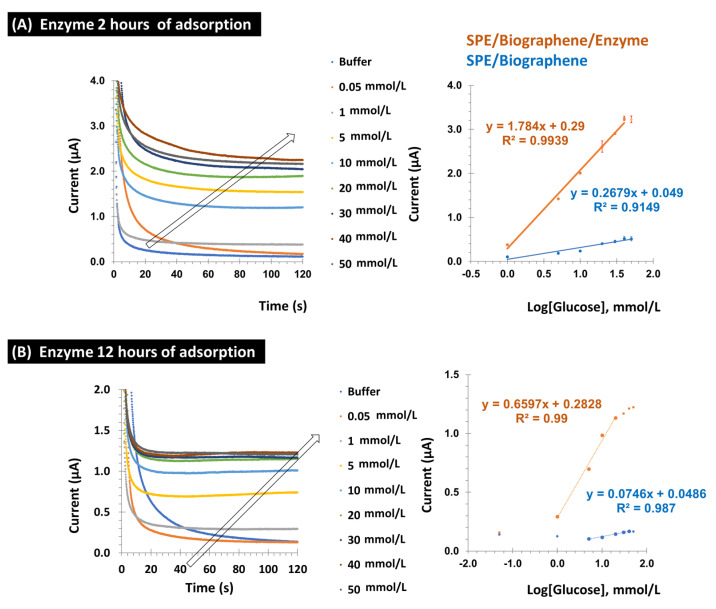
Calibration curves of the biographene sensor with the enzyme adsorbed on the Au-SPE surface during (**A**) 2 h and (**B**) 12 h. Amperometric response of the GOx/Biographene modified gold SPE to the successive addition of glucose at a potential of +0.6 V in PBS buffer 7.2 and 0.1 mol/L KCl in a concentration range of glucose (0.05–50 mmol/L).

Figure 4A shows the calibration curve for the modified Au-SPE electrode, both with and without the enzyme (which was used as a control), along with the corresponding triplicate error bars. The slope of the calibration curve for the enzyme-modified electrode was 1.784 ± 1.5 µA/log[Glu] mmol/L, while the slope for the electrode without the enzyme (control) was 0.2679 µA/log[Glu] mmol/L. This significant difference in slope indicates that the enzyme-modified electrode has a higher sensitivity to changes in glucose concentration. In particular, the enzyme-modified sensor is more responsive to fluctuations in glucose levels, making it more effective for applications requiring precise glucose quantification.

The linear range for glucose detection with the enzyme-modified electrode covered concentrations from 1.0 to 40 mmol/L. The precision of the sensors was assessed on the basis of the standard deviations of the measurements. For the Au-SPE/Bgr/GOx configuration, the standard deviation was less than 10.7%, while for the Au-SPE/Bgr configuration, it was even lower at less than 5.1%. These results show that both sensor configurations have good precision, with the enzyme-modified electrode offering higher sensitivity and stability in glucose detection.

To investigate the influence of the incubation time of the enzyme on the performance of the biosensor, the incubation time was extended to 12 h (Figure 4B). The calibration curves for the electrodes functionalized with GOx showed that the slope of the curve for the enzyme-modified electrode was higher than for the electrode without enzyme. Specifically, the slope for the enzyme-functionalized electrode was 0.66 µA/log[Glu] mmol/L, while the slope for the control electrode (without enzyme) was 0.075 µA/log[Glu] mmol/L (Figure 4B). The standard deviations for the Au-SPE/Bgr-GOx and Au-SPE/Bgr configurations were 3% and 2%, respectively, indicating good precision for both sensor configurations.

When comparing the slopes for the two incubation times (2 h vs. 12 h), the slope for the 12 h incubation was found to be approximately 37% lower than that for the 2 h incubation. This observation suggests that extending the incubation time of the enzyme beyond 2 h does not significantly improve the sensitivity of the biosensor. In fact, a longer incubation time appears to result in a decrease in the response of the electrode to glucose, suggesting that there is optimal incubation time and that a further extension does not improve performance.

#### 3.3.2. PBNCs-GO-Based Sensor

**Figure 5:**
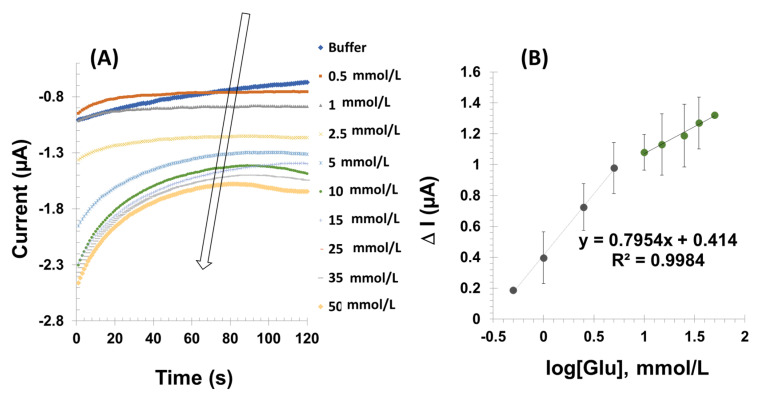
(**A**) Amperometric response of the GOx/PBNCs-GO modified gold SPE to the successive addition of glucose at a potential of 0 V in PBS buffer 7.2 and 0.1 M KCl in a concentration range of glucose (0.5–50 mmol/L). (**B**) Calibration curve in PBS buffer 7.2 and 0.1 M KCl in a concentration range of glucose (0.5–50 mmol/L).

Upon assembling the Au-SPE/PBNCs-GO-GOx modified electrode, it was observed that upon adding glucose, the current initially decreased, stabilizing after 40 s. Figure 5 displays the calibration curve, demonstrating a linear response to glucose concentration within the range of 0.5 to 50.0 mmol/L (R^2^ = 0.9984). The limit of detection was 0.33 mmol/L (calculated as 3 × SD/sensitivity, where SD is the standard deviation of the baseline current), with a slope of 0.7954 ± 1.5 µA/log[Glu] mmol/L.

When we compare the two approaches of Bgr and PBNCs-GOx based biosensors, the Bgr shows a higher slope of 1.784 ± 1.5 µA/log[Glu] mmol/L and a wider linear range. Subsequent studies focused on exploring the potential advantages of the Bgr-based biosensor.

### 3.4. Spiked Serum Samples Analysis of the Bgr-Based Sensor

The standard addition method was used to determine glucose levels in diluted serum samples, with concentrations ranging from 1.0 to 5.0 mmol/L glucose (Table 1). These assays were performed in triplicate, using CA measurements. Excellent agreement was observed between the amounts of glucose added and the amounts found, with recoveries ranging from 91.5% to 94.0%. The relative standard deviation obtained for the reproduced tests was less than 5.8%, while for the repeated tests it was less than 5%, reinforcing the remarkable precision of this new approach.

Thus, it was found that the highest maximum error was 8.5%, thus demonstrating its ability to detect glucose, even with the interference of other compounds present in the serum.

### 3.5. Proof of Concept of the Biofuel Cell Performance

**Figure 6:**
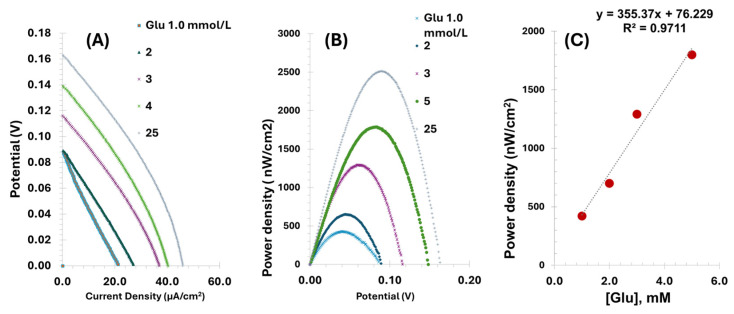
Polarization curves (**A**) and current curves (**B**) obtained with PBS solutions of Glucose with a concentration of 1.0 to 25 mM in PBS. The current density refers to the geometric surface area of the anode (0.064 cm^2^). (**C**) Current generated by the BFC.

The Bgr-GOx electrode was used as the anode in a membraneless miniature biofuel cell, while PBNCs-GO-GOx served as the cathode. No external mediators were added to the stock solution. The power and polarization curves for glucose concentrations between 1.0 and 25 mmol/L are shown in Figure 6. The polarization curves have a characteristic shape, starting with a small activation region, followed by a broad ohmic polarization region and ending with a sharp, steep section where the limits of mass transfer become clear. This behavior is typical of biological fuel cells where ohmic losses are the predominant factor at higher current densities.

With increasing glucose concentration, the polarization curves shifted upwards, with the OCP lying between +0.09 and +0.17 mV (Figure 6A). The corresponding power curves (Figure 6B) show a clear correlation between the power output and the glucose concentration. The maximum power output was 2.5 µW/cm^2^ at a voltage of +0.17 mV and a current density of 50 µA/cm^2^ at a glucose concentration of 25 mmol/L. Although the power output is relatively low, the fuel cell showed remarkable responsiveness to fluctuating glucose concentrations, emphasizing its potential as a self-powered glucose sensor.

After optimization, the biosensor showed a linear response to glucose concentrations in the range of 1.0 to 5.0 mmol/L, with a sensitivity of 355.37 nW·mmol^−1^·cm^−2^ (Figure 6C). This lower limit of the linear range is below typical glucose levels in healthy individuals, who generally have glucose concentrations between 3.8 to 6.9 mmol/L [44]. However, this range is suitable for monitoring glucose levels in patients with diabetes, whose glucose concentrations can rise up to 7.0 mmol/L or more in physiological fluids.

## 4. Conclusions

Our study presents a pioneering approach to glucose monitoring using an SPE-based glucose/oxygen fuel cell with a uniform substrate for both the anode and cathode electrodes. This novel configuration showed impressive reactivity over a range of glucose concentrations, eliminated the need for sample mediators, and exhibited reasonable response times. In particular, we achieved a maximum output power of 2.5 µW/cm^2^ at a voltage of +0.17 mV and a current density of 50 µA/cm^2^ at a glucose concentration of 25 mmol/L. Although these values may seem modest, they form a solid basis for future progress in this field.

Our results suggest that significant performance improvements in enzyme-based fuel cells can be achieved by optimizing electrode configurations, exploring a variety of nanomaterials and tailoring electrode surfaces. This shows that our method has great potential as a valuable tool for glucose monitoring in clinical and diagnostic applications. The simplicity of our approach has significant implications for the development of fully integrated analyzers for PoC glucose monitoring. By leveraging the innovative capabilities of SPE technologies, our work not only addresses a critical need in biomedical diagnostics but also lays the foundation for future advances with broader applications across the biomedical spectrum.

Furthermore, a biosensor able to work autonomously with an EBFC makes it ideal for remote or resource-limited areas. Its design can be adapted for the detection of different analytes, which expands its diagnostic potential. By using sustainable nanomaterials such as biographene, it supports the development of environmentally friendly technologies. Its affordability and portability improve access to diagnostics in underserved regions and combat inequalities in healthcare. In addition, a compact design and low power consumption make it suitable for integration into portable devices that enable non-invasive continuous glucose monitoring and advance personalized medicine.

## Figures and Tables

**Table 1:** Accuracy tests with biographene-based sensors with spiked serum samples.

[Glucose], mmol/L Added	Error (%)	Recovery (%)
1.0	7.5	92.5
2.5	6.0	94.0
5.0	8.5	91.5

## Data Availability

The original contributions presented in this study are included in the article. Further inquiries can be directed to the corresponding author(s).
